# Determinants of implementation of a stepped care intervention for adolescents and youth living with HIV in Kenya: a qualitative evaluation

**DOI:** 10.1186/s12913-025-12875-7

**Published:** 2025-05-15

**Authors:** Nok Chhun, Dorothy I. Mangale, Kawango Agot, Winnie A. Owade, Julie Kadima, Jacinta Badia, James K. Kibugi, Pamela K. Kohler, Grace John-Stewart, Kristin Beima-Sofie

**Affiliations:** 1 Department of Global Health, University of Washington, 3980 15th Ave NE, Seattle, WA 98195 USA; 2 Department of Oncology, Washington University, St. Louis, MO USA; 3 Impact Research and Development Organization, Kisumu, Kenya; 4 Department of Child, Family, and Population Health Nursing, University of Washington, Seattle, WA USA; 5 Department of Epidemiology, University of Washington, Seattle, WA USA; 6 Department of Pediatrics, University of Washington, Seattle, WA USA; 7 Department of Medicine, University of Washington, Seattle, WA USA

**Keywords:** Consolidated framework for implementation research (CFIR), Implementation determinants, Implementation science, HIV, Adolescents and young adults, Differentiated service delivery, Kenya

## Abstract

**Background:**

Differentiation of HIV services, a client-centered strategy, may improve care outcomes among adolescents and youth living with HIV (AYLHIV). Understanding health provider perceptions of barriers and facilitators that influence implementation can optimize adoption and sustainment of health systems interventions.

**Methods:**

The Data-informed Stepped Care (DiSC) study was a cluster randomized controlled trial of a stepped care intervention in 24 HIV care facilities in Kenya. At each visit, providers used an assessment tool to allocate AYLHIV to services according to level of need. Stable clients were allocated to differentiated service delivery (DSD) with less frequent visits. Intensified services, including behavioral counseling, were provided for those with greater likelihood of loss to follow-up, mental health issues, or viral non-suppression. We conducted focus group discussions (FGDs) with providers across 12 intervention sites between January-February 2023. FGDs used a semi-structured interview guide, grounded in the Consolidated Framework for Implementation Research, which were audio-recorded and transcribed. Transcripts were analyzed using a team-based rapid turnaround approach to characterize key determinants influencing adoption, reach, and fidelity.

**Results:**

Providers were enthusiastic about, and quick to adopt, the DiSC intervention. They found the DiSC tool easy to use and felt it provided a relative advantage by improving service delivery efficiency and prioritizing time with higher need AYLHIV. Providers noted the importance of tool flexibility to align with changing national guidelines. They expressed concerns about compatibility with existing workflows at facilities exclusively using electronic medical record (EMR) systems, suggesting EMR integration will be needed for intervention sustainment. AYLHIV eligible for DSD benefited from clinic visit intervals that aligned with the school calendar, which posed a challenge for reaching AYLHIV in more intensive steps. Provider collective efficacy was important in consistent implementation of DiSC and was facilitated by continuous quality improvement meetings, access to knowledge and information, and perceived intervention effectiveness. Supportive leadership was an important driver of implementation success, through availing space for mental health counseling sessions and time for client-provider interactions outside standard operating hours.

**Conclusions:**

Consideration of alignment with national policy, integration into clinic workflows, school schedules, and leadership engagement, will be important for sustained use of differentiated care interventions.

**Trial registration:**

ClinicalTrials.gov, NCT05007717. Registration date: July 13, 2021.

**Supplementary Information:**

The online version contains supplementary material available at 10.1186/s12913-025-12875-7.

## Background

Adolescents and youth living with HIV (AYLHIV) continue to bear a disproportionate HIV burden with lower retention and viral suppression rates when compared to adults [1–3]. A critical gap remains in reaching viral suppression targets for AYLHIV [4], with greater than 30% not virally suppressed after 12 months on treatment [5]. Achieving the UNAIDS 95-95-95 targets for AYLHIV will require interventions designed to meet their unique social, interpersonal, and developmental needs [6]. Client-centered strategies that have proven effective for adult populations, such as differentiation of HIV services [7], may also benefit adolescents and young adults.

Differentiated service delivery (DSD) models of care have been used in a variety of settings to improve treatment adherence, retention in care, and viral suppression [8, 9]. Recently updated World Health Organization (WHO) guidelines include adolescents and young adults as eligible for DSD [10]. In alignment with updated WHO recommendations, the Kenya Ministry of Health revised their HIV Prevention and Treatment guidelines to expand differentiated care services to AYLHIV in August 2022 [11]. As a result, AYLHIV are now eligible for services for which they did not previously qualify, such as multi-month refills of antiretrovirals or longer visit intervals between clinic appointments. As DSD services continue to expand to new populations, it will be important to evaluate strategies for implementing DSD alongside evaluation of the impact of DSD on HIV care and treatment outcomes [12–14].

The “Data-Informed Stepped Care (DiSC) to improve adolescent HIV outcomes” study was a hybrid type 1 cluster randomized controlled trial testing provision of a stepped care program including DSD and intensified services across 24 HIV care facilities in Kenya. Using routinely available data, a clinical assessment tool, and a stepped care service delivery approach [15], DiSC included intensified services (e.g., motivational interviewing, cognitive behavioral therapy sessions, enhanced adherence counseling) for higher need AYLHIV, in addition to differentiated services for AYLHIV who are effectively managing their care [16]. Examining the implementation of expanded service delivery strategies can provide timely and critical knowledge about how to optimize HIV care tailored for AYLHIV needs [6, 10].

In this qualitative assessment we aim to identify key barriers and facilitators that influenced adoption, reach, and fidelity of DiSC during early phase implementation. Additionally, we characterize providers’ experience delivering differentiated care services for AYLHIV and highlight recommendations for DiSC integration and scale-up across HIV care clinics in western Kenya.

## Methods

### Study design, setting, and population

**Fig. 1:**
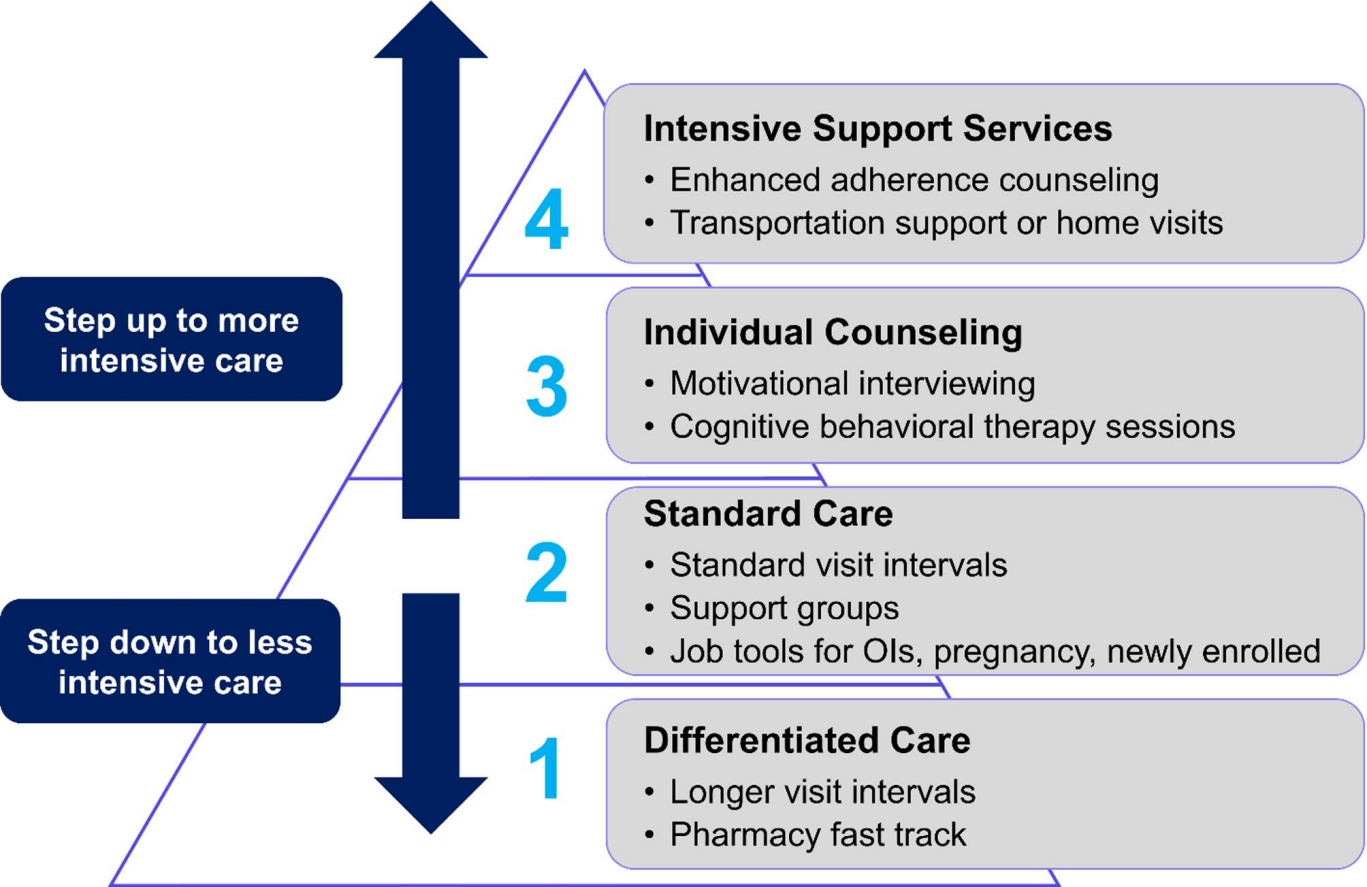
Data-informed stepped care (DiSC) intervention. DiSC is a multi-component intervention that uses a Stepped Care delivery approach along with a clinical assessment tool to assign adolescents and youth living with HIV to different steps of services aligned to their needs. OI, opportunistic infections

DiSC is a multi-component intervention where health providers (i.e., clinical officers or nurses) use a clinical assessment tool, informed by routinely available data, to assign AYLHIV, ages 10–24, to different steps of services (differentiated care, standard of care, individual counseling, or intensive support) aligned to their needs (Fig. 1). Services offered for those in Step 1 are aligned with DSD, with less frequent scheduled visits and fast-track pharmacy visits. In Step 2, AYLHIV are assigned to standard of care and receive standard visit intervals per Government of Kenya guidelines [11]. Intensity and frequency of services increase as AYLHIV step up; those in Step 3 receive cognitive behavioral therapy (CBT) counseling sessions if mental health support is needed. Lastly, clients with unsuppressed viral loads are assigned to Step 4 and receive enhanced adherence counseling sessions, transportation support to attend clinic appointments, or home visits. A more detailed description of DiSC has been previously published [16]. Clinical officers, nurses, or adherence counselors delivered the services as part of their role and responsibilities at the HIV care facilities. However, a subset of providers who participated in the DiSC trial were trained to deliver CBT sessions as part of the intervention, along with adherence counselors. The training consisted of one week of didactic instruction and systematic feedback, with mentored supervision of those trained for the trial duration.

The DiSC study was conducted in 24 HIV care facilities across Kisumu, Homabay, and Migori counties from April 2022 to August 2023 [16]. The healthcare facilities located in the three counties represented a mix of urban, rural, and fishing communities. HIV prevalence reported for these counties was 18.5% for Homabay, 17.5% for Kisumu, and 11.8% for Migori, compared to the national average of 4.5% reported in 2020 for adults aged 15–49 years old [17]. The highest rates of HIV acquisition among youth ages 15–24 years are concentrated in these high prevalence counties in western Kenya [17].

### Theoretical framework

We used the Consolidated Framework for Implementation Research (CFIR) [18, 19] to examine barriers and facilitators experienced during early phase implementation of DiSC. The semi-structured interview guide (see Supplementary Material 1) used during discussions with providers was grounded in relevant CFIR constructs across five domains: intervention characteristics (e.g., complexity, relative advantage), inner setting (e.g., compatibility, access to knowledge and information), outer setting (e.g., patient needs and resources, external policies and incentives), characteristics of individuals (e.g., knowledge and beliefs about the intervention), and process (e.g., engaging, executing), with an additional domain (characteristics of systems) and novel constructs (e.g., resource continuity, collective efficacy, team characteristics, community characteristics) relevant in low- and middle-income settings [19].

### Data collection

Between January and February 2023, we conducted 12 focus group discussions (FGDs) with providers from DiSC intervention sites (Kisumu *n* = 3, Homabay *n* = 4, Migori *n* = 5). All health providers from intervention sites who participated in continuous quality improvement (CQI) meetings to optimize DiSC implementation at their HIV care clinic [20] were purposely recruited for participation in FGDs. Providers were ≥ 18 years old and were nurses or clinical officers with experience delivering HIV care services to adolescents and young adults.

Discussions were facilitated by an experienced Kenyan social scientist (WAO) who was not involved in study implementation and did not know the participants prior to the FGDs. FGDs were also supported by two additional research team members, who were not involved in providing support supervision during the CQI meetings (JK and NC). Discussions were primarily conducted in English, but providers were also encouraged to use Kiswahili to describe their experiences if preferred. FGDs had a median of 3 providers (interquartile range [IQR]: 3, 4), lasted a median of 75 min (IQR: 60–86 min) and were audio recorded, translated as needed, and transcribed verbatim. Structured debrief reports were generated by the notetaker (NC) within 48 h to summarize key information from each FGD.

### Data analysis

A *Sort and Sift*,* Think and Shift* rapid qualitative analysis approach [21–23] was used to identify barriers and facilitators of DiSC intervention implementation, and provide recommendations on integration and scale-up. Transcripts were analyzed using the following adapted team-based rapid turnaround qualitative analysis approach. First, an FGD summary template was developed using the CFIR constructs and implementation outcome measures organized by three main headings: facilitators, barriers, and recommendations. Three team members (NC, DIM, KBS) summarized one common transcript using the summary template to ensure consistency in capturing key themes and constructs influencing DiSC adoption, reach, and fidelity during the early implementation phase. Team members discussed and compared summary styles and revised the transcript summary to improve usability. Afterwards, two team members (NC, DIM) summarized an additional common transcript using the revised template summary, which was then reviewed by a third team member (KBS). Once consistency was established, the remaining transcripts were divided between two team members (NC, DIM) and each transcript was summarized by one team member and the transcript summary reviewed by another member. During transcript summary review, disagreements in identified constructs were noted and resolved through group discussion with the larger qualitative analysis team. One primary analyst (NC) abstracted data from final versions of transcript summaries into an excel matrix that displayed the data by site, CFIR constructs, and barriers and facilitators to adoption, reach, and fidelity. Identified constructs were compared between sites to summarize their overall influence on implementation. The matrix and preliminary results were shared and discussed with local research team members (WAO and JK) who were present during the FGDs to ensure agreement with main findings and overall synthesis.

Descriptive characteristics of providers were summarized using counts, proportions, medians and interquartile ranges (IQR) using the R statistical computing environment [24].

## Results

A total of 43 health providers participated in 12 FGDs. The majority of providers were clinical officers (72%), male (51%), with a median age of 34 years (Interquartile range [IQR]: 31–37). Providers reported a median of 4 years (IQR: 1–7) working at their respective clinic, and a median of 6 years (IQR 5–9) engaged in providing care and treatment for AYLHIV (Table 1).

**Table 1:** Demographic characteristics of focus group discussion (FGD) participants (*N*=43)

Characteristic*	N (%) or Median [IQR]
Primary work location	
Comprehensive Care Clinic	39 (91)
Other^1^	4 (9)
Sex	
Male	22 (51)
Female	21 (49)
Age (years)	34 [31, 37]
Highest level of education: University/college	43 (100)
Healthcare provider classification	
Clinical Officer	31 (72)
Nurse	11 (26)
Nurse Counselor	1 (2)
No. of years at current clinic	4 [1, 7]
No. of years providing HIV care to PLHIV (all ages)	7 [5, 9]
No. of years providing HIV care to AYLHIV (ages 10-24 years)	6 [5, 9]

*Abbreviations*: *PLHIV* People living with HIV, *AYLHIV* Adolescents and youth living with HIV

*Majority of health providers filled out a demographic survey prior to the start of DiSC trial implementation, with 6 providers filling out demographic information post-FGD

^1^Other work location includes vertical transmission, maternal child health, and outpatient departments

Overall, providers described high acceptability and a general positive experience engaging with the DiSC intervention. They were enthusiastic about how the clinical assessment tool guided service provision, perceiving an improvement in client satisfaction by using this service delivery strategy.

*“I really like using the tool*,* because it’s really guiding whether to step up*,* step down or maintain the client on the current care that the client is [receiving]. And even our clients*,* actually*,* when we offer them these services using the tool*,* they’re getting satisfied. And when you serve a client and the client is satisfied*,* you as a healthcare provider also feel satisfied. I say it has really helped us and our clients.”* – [P2, Facility 4]

An overview of barriers and facilitators influencing adoption, reach, and fidelity during early phase implementation of DiSC are presented in Table 2. Key determinants were predominantly related to intervention characteristics, inner setting, and outer setting CFIR domains.

**Table 2:** Health provider perceptions of determinants influencing adoption, reach, and fidelity of a stepped care tool to support service delivery for adolescents and youth living with HIV

CFIR* domain	CFIR Construct	Description	Illustrative Quotes
*Facilitators*			
* Adoption*			
Intervention characteristic	Design quality and packaging	The tool is comprehensive and designed to cover relevant topics	“[T]he tool is short and precise. And it has touched like everything. Because there [are] elements of depression here. There’s [the] element of opportunistic infections, and there are elements of breastfeeding or pregnant mother[s]. And then there’s this step that the client is supposed to be assigned to… So you can’t miss, you can’t miss anything.” – [P3, Facility 12]
Intervention characteristic	Complexity	The tool is easy to use and provides guidance on what to do during the client visit	“[W]e want to appreciate the tool because the tool is not that complicated. Actually, the experience that we got from the first time is that it even made our work easier. It was more simplified, because without the tool we could just talk and ask anything but at least the tool is guiding us.” – [P2, Facility 11]
Inner setting	Readiness for implementation: Access to knowledge & information	Training provided information needed to implement the tool	“[T]he training was good generally and it helped us to do the right thing. Yeah, so currently we don’t have any challenge in stepping our clients. Yeah, it was excellent is what I can say.” – [P3, Facility 7]
Inner setting	Collective efficacy	CQI meetings facilitated collaborative problem solving to address challenges	“[W]hen we started using the tools, that’s why we were evaluating every two weeks to see where we have had the gap or the challenges and at each point we were giving suggestions and then implementing them… we sat, as a team in our discussions…” – [P4, Facility 4]
* Reach*			
Intervention characteristic	Adaptability	Providers were able to adapt the intervention to meet client needs	“It depends on case-by-case, one-on-one…….Some will come here to date, some might call, depending on the distance. Like the other one I’m managing is in [location], the other one is a form 3 class, so I can’t allow him to be coming here. So sometimes they come over the weekend, sometimes we talk over the phones.” – [P6, Facility 10]
Outer setting	Patient needs and resources	Providers were able to adjust the clinic appointment schedule to accommodate the school calendar	“[T]he dates we were giving were not matching with their school calendars…So, we realized that and we were able to change. Now we are……giving dates according to school calendars. This helps us because we could now not get missed appointments.” – [P1, Facility 2]
* Fidelity*			
Intervention characteristic	Perceived intervention effectiveness	Providers observed a positive impact of the intervention on clients	“The adolescents, at first they were not opening up. But through knowledge we got on how to probe the client, you probe, you be friendly with them, then you talk to them well. You are in that age of theirs. And then they just open up and now be your friend [and] tell you everything….Now when they come, they want to see you because the way you are talking to them. I think that it has helped a lot.” – [P1, Facility 2]
Inner setting	Collective efficacy	Providers problem solved to optimize their ability to provide services	“[A]t some point we had some adolescents which had no valid viral loads. We had to request [them from] the office and they tried their level best and provided us with some viral load tubes, which they brought, and we were able to draw blood for the missing adolescents. And through that….we managed to get some valid results for those adolescents which helped us in managing them.” – [P3, Facility 2]
*Barriers*			
* Adoption*			
Intervention characteristic	Design quality & packaging	Language in the tool was complicated, which made it challenging for providers to explain to clients	“So, my concern for the tool is about the step three; over the last two weeks, how often have you been bothered by the following problems? So, the question can be posed very well in English to a patient, but now level of understanding is a problem. So, you need to translate this to either Kiswahili or Luo for the patient to understand. So, I find it quite challenging even to translate it for someone to understand….” – [P2, Facility 6]
Inner setting	Readiness for implementation: Access to knowledge & information	Some providers felt that refresher trainings would have been beneficial	“It is a good thing and even as we proceed even refreshers are needed so that we can really be sure that whatever that we are doing on the ground are the real thing…, yeah so because these are real clients and it needs frequent practice. Yeah, it needs frequent practice for you to master so that you are able to handle. – [P1, Facility 5]
* Reach*			
Intervention characteristic	Design quality & packaging	Availability of training tools in another language will support providers in assessing mental health	“[I]n the training, it is in English but now it is upon you now to translate to the local language that the client can understand. So now as you are transferring, you may miss and say something totally different than was meant there.… Those clients, maybe they are not up to date. Like if you start talking in English, they may not understand well or maybe they may not respond well, so it is upon you to turn [it] into another language, Kiswahili or any other language that you can fit in.” – [P1, Facility 5]
Outer setting	Community characteristics	Challenges with assessing mental health for younger AYLHIV	“[T]he younger ones, you have to interrogate probe them. That’s why mostly we want the younger adolescents to come with their guardian or parents. Because for example, if your child has lost some interest or [is] depressed with him or her, she will not know. But the parent or guardian will know. Or the teacher will know, because maybe the patient or the client was doing well in class.” – [P2, Facility 2]
* Fidelity*			
Intervention characteristic	Design quality & packaging	Availability of training tools in another language will support providers in assessing mental health	“[T]he aspect of mental assessment can only apply on those older adolescents. For those very young adolescents, like let’s say below 15 years….they may not understand so well. So the issue of mental assessment….I don’t know how it can be simplified so that also the very young adolescents can understand….” – [P3, Facility 1]
Inner setting	Compatibility	Not aligned with ART guidelines	“There is need to [make sure], as we are designing this tool, the designers are up to date with the guideline because you see for us the guidelines reign supreme. Should there be a conflict between any document and the guideline, the guideline carries the day [Laughter]. So that may create a bit of friction because now you don’t know whether to proceed with the tool or revert back to the guideline. So the default setting is the guideline. So the designers should just make it in a way that it does not contradict or be seen to be antagonizing the guideline.” – [P6, Facility 9]
Inner setting	Readiness for implementation: Available resources	Viral loads needed for step assignment were not readily available	“[T]he tool came about at a season when the facility was also not able to take samples for viral loads. So being able to administer the tool….majority of them could not be assigned properly because of the missing key interventions that would inform the tool.” – [P1, Facility 8]

*Consolidated Framework for Implementation Research (CFIR)

### Implementation outcome: adoption

Primary drivers of adoption included health provider access to knowledge and information, perceived complexity of implementation, and shared belief in their collective efficacy. Providers perceived that their readiness for implementation was facilitated by the quality of training they received prior to DiSC implementation. The majority of health providers felt that the training was informative and gave them the knowledge needed to do what was “right” for their clients.

*“The training was sufficient so that it prepared us to face the challenges directly from the clients. If it were that we were not trained*,* then it will be difficult to lend information to the clients because it’s me and my colleagues the clients were questioning. So*,* if we’re not trained*,* then it could be difficult to respond. So*,* the training was sufficient*,* it prepared us to face the work.”* – [P5, Facility 4]

In contrast, some providers reported gaps in the quality of the training sessions noting that the training was too short for them to absorb all of the information or led them to mistakenly believe that the DiSC tool would be complicated to implement. Additionally, although providers were trained on *how* to use the tools, some felt they did not leave the training with an understanding of *why* they were assessing and assigning AYLHIV using the DiSC tool. However, once they began using the tool, they found it to be valuable in addition to decreasing their workload.

*“[A]t first*,* it was like this is another difficult one… we were saying why are these people adding us more jobs*,* we have a lot of work and you are adding more. So*,* the fact still remains that at the beginning we were not interested… which I think as time goes*,* that one faded out and we noted that in fact*,* it even lessened the work at the end of the day.”* – [P2, Facility 3]

With time, providers’ perceived complexity of the intervention improved, it became easier to implement, and they found the overall design and service delivery approach beneficial. They described the DiSC intervention as providing a relative advantage when compared to existing service delivery strategies because it improved efficiency by prioritizing time with higher need AYLHIV while simultaneously reducing time spent with AYLHIV who were doing well. This perceived advantage further facilitated provider adoption.

*[T]he tool has really reduced our work; once the clients who are categorized as established; we will now give them a longer TCAs [clinic appointments] so we don’t see so many clients with their specific day…. Initially we used to see more than 70 clients in one week*,* but currently with the Step* [1] *we can give very long TCAs so we see like 30*,* 20*,* it has really reduced our workload.”* – [P2, Facility 12]

Providers were motivated to continue implementing DiSC due to perceived intervention effectiveness. They noticed the impact of DiSC on clinical outcomes and recognized that AYLHIV can benefit from the differentiated service delivery model.

*“I think our intention was to see*,* can it really work? And I can see that it is working. Because when we are saying to them*,* Step 1*,* and they’re still adhering*,* they’re still keeping their TCAs [clinic appointments] and they’re still virally suppressed. We’ve not seen any challenge by putting them in Step 1.”* – [P2, Facility 4]

Providers at one facility were so enthusiastic about their experience during implementation that they integrated DiSC into their workflow for all AYLHIV at their clinic, irrespective of enrollment in the clinical trial, and considered the tool a routine “part of their practice.”

*“[W]e decided now*,* despite the fact that the number which the study wanted is over*,* but our clients were not over*,* remember we are offering services to clients*,* so we took initiative as [a] facility and now we are using it at our desk*,* that is the clinician desk*,* so he is able to step at that point.”* – [P2, Facility 3]

Providers’ participation in continuous quality improvement meetings promoted their adoption of DiSC. During these meetings, providers identified the challenges they experienced during early phase implementation and the targeted adaptations they made to optimize DiSC implementation to their context, thereby increasing their shared belief in the collective capacity to use the clinical assessment tool and deliver the best services to AYLHIV. Further supporting their collective efficacy was the culture of teamwork and leadership engagement at their respective facilities. Providers felt that the relationships across departments, and collaboration among a diverse team of staff and other health cadres helped them to achieve implementation goals.

### Implementation outcome: reach

Patient needs and resources, design quality and packaging, adaptability, and community factors influenced AYLHIV reach and engagement with the DiSC intervention. Providers perceived that DiSC facilitated individualized adolescent care to better meet adolescent needs.

*“[T]his DiSC tool has assisted us in attending to adolescents individually and not as a group as we used to do. With the introduction of this tool*,* we were able to identify those who had specific needs…At first*,* we lacked confidentiality and privacy. We had no specific room for that. With the introduction of the tool*,* we were able to create a room for that. And now more adolescents were able to open up. And through that we were able to get a lot of problems which these adolescents go through*,* and we were able to advise them accordingly. Though we were not able to manage them all because some were beyond us*,* but at least those that were within us*,* within our reach*,* we were able to tackle them. So*,* I can say that this tool has really helped us.”* – [P3, Facility 2]

Providers observed that the design quality and packaging of DiSC supported client reach and engagement. Providers found that the longer appointment intervals relieved the transportation burden for adolescents who were located further away from clinics, allowing them to more consistently make scheduled appointments. For example, AYLHIV eligible for Step 1 differentiated care services benefited from clinic visit intervals that were aligned with the school calendar, which had been identified by providers as a primary reason why AYLHIV in boarding schools missed appointments in the past or sent their treatment supporters or caregivers to the clinic instead.

*“…if you really go through the DiSC tool*,* it provides a kind of patient centered support. Because by stepping the clients*,* it’s not just about assigning them TCAs [clinic appointments] because it makes you realize each and every adolescent is unique in their own way… So even if they’re going to boarding school*,* and the majority of the boarding schools nowadays*,* they don’t go past three months*,* so if I give them drugs for three months*,* what will make them miss their appointments? They will not have a reason. And then it tends…it works as a kind of motivation for them.”* – [P1, Facility 8]

The adaptability of DiSC made it possible for providers to vary implementation approaches to maximize reach and engagement for AYLHIV. Providers described situations where leadership supported the changes in workflow necessary to meet AYLHIV needs, including accommodating visits that were outside of regular hours.

*“[T]here are some occasions where maybe you’ve realized that the client or the adolescent or the caregiver is not able to make it maybe during the weekdays. So*,* you’ve realized that maybe through a local arrangement*,* maybe this client can get time during the weekend? So*,* by this*,* I want to say that the administration did come and supported us because without them giving us that opportunity to be here on a non-working day then it means that it could not work. So that administration did support us because we could come even during the weekend and attend to those one or two unique cases.”* – [P2, Facility 11]

Provider experience during DiSC implementation led to their recommendation of age-specific strategies to support AYLHIV with retention in care. Providers felt that younger AYLHIV need interventions focused on caregiver engagement whereas older AYLHIV need interventions focused on client motivation to support them with independently managing their care as the role of caregiver engagement changes.

*“… [W]hen our adolescents come for their scheduled clinic*,* what we used to tell them is that the support will not continue*,* probably it might not be there in the near future. So*,* the issue here is just a matter of preparedness for the sustainability*,* the support might not be there. So*,* they get prepared.”* – [P2, Facility 7]

For clients who were unable to come to clinic for various reasons, providers had the option to deliver intervention components, such as mental health counselling, over the phone, which reduced clients’ need to travel to the clinic for more intensive services in Steps 3 and 4. Despite options for alternative delivery, most providers preferred in-person interactions and believed their clients also preferred in-person services. Providers noted that delivery by phone was not ideal and did not allow them to observe AYLHIV responses and emotional state.

*“….[T]he hard cases we have to meet the client face to face the reason being sometimes we are doing over the phone you know other people may be in school. Yeah*,* they may be in a place that they may not have privacy now to talk things so you may not know whether you are dealing with the real thing or they’re just saying things to please you so that you understand.”* – [P1, Facility 5]

Providers found it challenging to implement some of the mental health components given the lack of community recognition of the importance of mental health as a health issue. Lack of information and awareness made it hard to engage and follow-up with adolescents who were stepped to receive cognitive behavioral therapy sessions.

*“The mental issues thing in our African setting. They don’t believe it’s an illness. So it is you to fix time with this client. In most cases*,* they are like*,* you are bothering them a lot….The issue is getting this client*,* like for the client*,* I’m supposed to do “think in a different way part two*,*” [a session in the mental health counseling series] now she’s even not within*,* I’m still tracing her.”* – [P1, Facility 3]

### Implementation outcome: fidelity

Consistent implementation of DiSC per protocol was driven by stakeholder engagement, perceived intervention effectiveness, workflow and guideline compatibility, and existing human and material resources. Multiple levels of stakeholders were identified that had key roles in supporting implementation, including AYLHIV, and their caregivers, providers, and facility leadership. Providers described how leadership support was important during implementation.

*“Administration support*,* because of the time we take*,* you know*,* sometimes*,* you’re not supposed to take long with those clients*,* because of ABCD. But now through the administration*,* and you explain to the clients*,* they allow those clients to be here fully. And then maybe sometimes you need to be here longer you’ll be supported to be here. And then maybe you want to be with that client. And there’s another thing that you should be doing. So*,* you are allowed to do*,* to continue with the stepping*,* and then another person takes over what you’re supposed to do.”* – [P4, Facility 11]

In addition to their influence on adoption, providers’ perception of intervention effectiveness and collective efficacy facilitated consistent implementation of DiSC throughout the early implementation phase. Perceived positive impact on AYLHIV outcomes encouraged consistent intervention use. While they reported consistently using the DiSC stepping tool with all AYLHIV, providers recognized that it was challenging to assess mental health among younger clients using the Patient Health Questionnaire (PHQ-2) [25], potentially leading to inaccurate stepping and misalignment of service provision with identified need for younger AYLHIV. As a result, they recommended the tool be revised to capture caregiver input as a way to better assess the mental health needs of younger AYLHIV.

*“I felt they were not the right indicators to assess mental health for that age. One*,* that children will never lose interest or will never have interest because they’re directed to do the normal activity*,* be it learning. Like in our context in Kenya we don’t develop interest in doing things even fetching water*,* cooking. We don’t develop interest; we are instructed*,* we are given direction. So*,* they have never developed interest to maybe in any of those activities you want to assess*,* they’re always instructed…. So those indicators I found them not age appropriate to assess for mental health.…That’s why maybe we did not get much of mental health when those cases are there. We missed them because of the questions.”* – [P6, Facility 6]

Related to implementation fidelity, providers identified compatibility with guidelines and workflows as the most influential challenge experienced. HIV care clinics were either fully paperless and using electronic medical records (EMR) or were in the process of transitioning. As a result, providers would sometimes forget to use the paper-based clinical assessment tool. However, they were able to collectively identify solutions to ensure that clients were being exposed to the tool and assigned to the correct ‘step’ when they returned to clinic.

*“[W]hile you are using the step tool*,* we need to work within the guideline that is universal in the entire country. So that was the biggest thing that we were seeing…also another thing that we see is we’re using the Kenya EMR [electronic medical record]; we are paperless but now the step tool was in paper form so you could easily forget once you’ve cleared the clients now you want to come and write on the papers.”* – [P2, Facility 12]

Providers found implementing the tool as designed difficult because viral load assessment cut-off measures were not aligned with updated MoH guidelines, a change which occurred during the study implementation timeframe. Furthermore, when viral load information needed for stepping was not available, providers were not able to assign AYLHIV to their correct ‘step’ which prevented AYLHIV from receiving services aligned to their needs. For example, AYLHIV who may have qualified for a lower step assignment, continued to receive more intensive services (e.g. Step 4 adherence counseling) placing a burden on the AYLHIV as well as reducing efficiency in prioritization of provider time and resources.

*“Like maybe when the viral load is not being done*,* like the experience we had; see like the whole [of] Kenya*,* the viral loads were not there. Now we’ll keep on stepping this person like maybe the previous viral load was just more than 50. So*,* this client will be always step four*,* step four…”* – [P4, Facility 11]

Additional challenges to fidelity were related to available resources. Once viral load results were available, there were delays in uploading results into the EMR, making them unavailable for point of care use. To overcome this challenge, providers described working as a team to update viral load information in the EMR themselves so that “at each and every time we have the current viral load,” to assign AYLHIV their correct step assignment.

*“…just to add on the viral load*,* another challenge we had was that given the long period that we’re not taking viral load*,* there was a backlog also*,* and in our facility*,* we only have one lab man. So*,* downloading of the results and keying them in the EMR [electronic medical record] used to take quite some time. So*,* at times also we had cases whereby the adolescents are seen with the previous viral loads yet there was a current one but has not been keyed in yet.”* – [P4, Facility 4]

### Recommendations for scale-up

Based on their experiences with delivery, providers recommended expanding implementation of the DiSC intervention in HIV care clinics across Kenya.

*“Let me say that whoever came up with this idea*,* it was a good thing. And for your information*,* it’s going to save quite a lot of adolescents. It’s going to save lives. So*,* something which is saving lives*,* what are you going to do about it? You roll it out*,* you expand it so that it captures a wider area of many youths to benefit.”* – [P3, Facility 3]

When considering scale-up within Kenya, providers outlined specific considerations to improve implementation and effectiveness (Table 3). First, providers recommended modifications to the DiSC clinical assessment tool that would inform care and management, such as categorizing AYLHIV by age groups to address differences among those moving into adolescence and those who are aging out.

*“I’d wish that the tool can be categorized to capture some of the uniqueness in those who are just stepping into adolescence and those people who are mature*,* almost getting out of adolescence… I would wish that we categorize them*,* so we have a tool specifically for maybe 15 to 24. And maybe 10 to 14. With some relevant questions in each age group to enable us to capture almost everything.”* – [P2, Facility 3]

**Table 3:** Recommendations for scale-up and optimization of DiSC implementation across HIV care clinics in Kenya

Themes	Description	Illustrative Quotes
Modifying the DiSC clinical assessment tool	• Categorize AYLHIV by age groups in order to address differences among those stepping into adolescence and those stepping out • Include missed doses as an indicator of adherence rather than a missed visit • Categorize opportunistic infections by level of severity to inform care management	*“[W]e have opportunistic infection. They’re staged*,* we have stage one*,* stage two*,* stage three*,* stage four. So*,* if you generalize it like the opportunistic infection*,* ideally it doesn’t bring the picture because even with us*,* if we see a client with maybe herpes*,* we stage that one as stage two*,* we are not much concerned too much to this client like a client with cancer*,* or a TB client.”* – [P1, Facility 1]
Implementing strategies to support DiSC integration into routine care and workflows	• Align criteria for step assignment with Kenya Ministry of Health National Guidelines • Incorporate paper-based DiSC clinical assessment tool into the electronic medical record (EMR) system • Integrate the DiSC clinical assessment tool with the Clinical Encounter Green Card, a form that is available in the EMR • Replace PHQ-2 mental health assessment questions with the PHQ-9 because it is what clinics are currently using • Develop reporting tools to monitor progress and track key milestones and outcomes	*“I think the tool needs to be updated as per the guideline so that it makes it easy for*,* for the clinicians and even the nurses who are implementing the tool as you roll it out to be able to not have two contradicting messages in the file. So that*,* you know*,* if the cut off for high viral load is 200*,* then it’s uniform regardless if it’s the tool*,* because the tool is supposed to support to give patients centered care. So*,* changes should be made same as in the guideline.”* – [P1, Facility 8]
		*“[S]taffing for the ministry if the tool is not integrated within the tools that are being used*,* they might find it difficult to implement…[W]e have the green card that is used to most of the government facilities. So*,* if it is added in the green card*,* then it will be easy for them to use because now*,* they’ll be going step by step.”* – [P4, Facility 4]
Building technical capacity to support scale-up and implementation	• Invest in human resources • Expand training to additional healthcare worker cadres, including adherence counselors, peer mentors, community health workers, and lab technicians • Provide support supervision • Cascade mental health training to all providers • Utilize train-the-trainer mentorship model	*It’s about sensitization*,* and the training and all that and I think that tool is so easy and it can be used by any other person after they undergo some kind of training.”* – [P2, Facility 11]
		*“[A]s we are going to escalate this thing into other facilities for us who have been trained you can make as a mentor we mentor other people so that if we mentor other people from other facilities the tool will be used optimally.”* – [P2, Facility 12]
Engaging stakeholders to ensure sustainability and scalability	• Preserve institutional knowledge in the face of turnover by involving and sharing information across all levels of the organization • Engage facility leadership • Engage clients in their own care • Involve caregivers in the care of AYLHIV • Involve external partners who are involved in HIV care and management • Engage multiple stakeholders at the subcounty and county levels during scale up • Engage with networks and organizations involved with AYLHIV, for example, teacher networks and organizations that advocate for individuals living with HIV • Foster community engagement	*“[Y]ou know*,* these adolescents*,* they come from the communities*,* and in our communities*,* we have allocated them*,* community health workers*,* who also works closely with them at the households*,* so maybe they would have also been involved*,* because they also do adherence monitoring at the households.”* – [P4, Facility 4]

Second, providers identified strategies to support DiSC integration into routine care and workflows that addressed modifiable barriers experienced during implementation. These included aligning viral suppression cut-off criteria with changing Kenya national guidelines and incorporating the paper-based DiSC clinical assessment tool into the EMR system and integrating it with the Green Card, a Ministry of Health Clinical Encounter form that provider’s currently fill out at every visit. Recommendations also centered around building technical capacity to support scale-up, including expanding access to knowledge and information by training additional healthcare worker cadres, including peer mentors, community health workers, and lab technicians, because “everybody at one point is involved.”

To ensure resource continuity during scale-up, providers supported cascading mental health training to other colleagues. This would address challenges experienced during DiSC implementation when providers who were trained to deliver the mental health sessions were transferred out to another facility.

*[N]owadays mental health is at a high rate and maybe what if the one who has been trained is not around. It will mean the client will not even get quality services.”* – [P4, Facility 5]

Lastly providers highlighted the importance of continued engagement with multiple key stakeholders, including AYLHIV, caregivers, health facility leadership, educators, external partnerships, networks and organizations, the community, and the Ministry of Health, for successful scale-up.

## Discussion

This qualitative evaluation aimed to identify key facilitators and barriers that influenced implementation outcomes of adoption, reach, and fidelity during early phase implementation of DiSC, a stepped care intervention for AYLHIV that was delivered in 12 intervention sites in western Kenya. We applied the CFIR, a determinants framework, to organize our understanding of key influences impacting implementation. Providers perceived that in Kenya, differentiation of HIV services was aligned with the school calendar for those in boarding school, and clients were able to manage their care with less provider interaction. Providers also perceived that under the DSD model available in the study, AYLHIV were maintaining their adherence, clinic appointments, and viral suppression. Our findings spotlight opportunities for strategy development to optimize integration of new interventions, such as DiSC that incorporate elements of DSD and other client-centered HIV services like psychosocial interventions for addressing AYLHIV mental health needs. Our intervention is novel in that the stepped care approach targets AYLHIV established in care, and those vulnerable to loss to follow up, by aligning available services to their individual needs.

Implementation of DiSC occurred against a background of policy recommendations that expanded DSD to adolescent and young adult populations. Alignment of DiSC study implementation timelines with changing guidelines offered an opportunity to assess how DSD for AYLHIV could be implemented in practice by providers, e.g., screening for eligibility and delivery of DSD services. Our study highlights the benefit of job aids and other tools that support providers in delivering care aligned to client needs. In our study, providers used the clinical assessment tool to guide use of routinely available data to place eligible AYLHIV in less-intensive differentiated services like longer visit intervals or pharmacy fast-track visits, while identifying those that need more targeted support. In research settings, various differentiated models of care have been successful in improving outcomes for AYLHIV, including retention in care [26, 27] and viral suppression [28]. An example of one approach to DSD in Kenya that has proven effective is the “Red Carpet Program,” where clients are fast-tracked to receive health services within a clinic setting [27, 29].

The perceived effectiveness of the DiSC intervention had a cascading impact on provider adoption of the clinical assessment tool and their consistent implementation of DiSC for supporting the care and management of AYLHIV. Provider recognition of improved service efficiencies as a result of the DiSC intervention also facilitated their adoption. Providers found that the longer visit intervals and fast-track ART refills that were part of DSD model used in DiSC, lessened their workload by staggering client return to clinic schedules and optimized time with clients who needed more support to stay in care. This finding corroborates evidence that differentiation of care enables health system reallocation of resources to better serve client needs [30, 31].

Fostering positive perceptions of the intervention was provider participation in CQI meetings during early phase implementation to support fit of the intervention with the complex ‘real-world’ context of the healthcare facility in which it was being implemented. As a result, a team-based learning environment may have promoted collective efficacy, a shared belief in provider capacity to achieve implementation goals. This finding is aligned with existing literature demonstrating that team-driven approaches, such as CQI, improve health service provision as well as client outcomes [32, 33].

Despite challenges experienced during implementation, including aligning viral suppression cut-off criteria with changing Kenya national guidelines and country-wide issue of unavailability of viral load data to inform care and treatment, providers recommended scale-up of the DiSC intervention. Provider recommendations for expansion of DiSC to other healthcare facilities in Kenya were informed by their experiences during implementation. Providers indicated that their readiness for implementation was facilitated by the training they received and recommended that mental health training be expanded to additional healthcare providers not trained during DiSC implementation. Although providers recognized the benefit of psychosocial interventions (i.e. cognitive behavioral therapy) for adolescents and young adults experiencing mental health challenges, an important barrier that emerged from the analysis was that the community was not sensitized to mental health issues and the seriousness of these challenges, which may impact AYLHIV demand for and experience with mental health service. Further research is needed to address community perceptions of mental health to promote environments that can support the overall well-being of AYLHIV. Mental health screening tools also need validation for the context, population, and local communities in which they are being applied. Provider challenges with assessing the mental health needs of AYLHIV during the study highlight the importance of validating tools to address potential cultural and linguistic barriers that may impact the accuracy of measurement, especially among younger adolescents.

Our study had several strengths. We used a team-based rapid turnaround data analysis approach to summarize and synthesize DiSC implementation experiences across different HIV care facilities in Kenya. Our application of CFIR to a low-middle-income country (LMIC) setting further expands the use of this determinants framework in global health settings and supports comparison with findings from other studies conducted in LMICs. Our study generated timely information about how to screen AYLHIV for DSD eligibility and deliver differentiated care using a data-informed approach. Lessons learned from our experience are generalizable to other settings interested in routinizing DSD for AYLHIV, now that policy barriers are no longer in place, and DSD is available outside of a research environment.

Our analysis has limitations. While the CFIR framework elucidated multiple determinants that affect implementation outcomes, it was difficult to characterize the influence of one determinant on another and map the synergistic relationships among determinants that impact implementation [34]. Additionally, this qualitive evaluation of early phase implementation is assessed from the perspectives of health care providers delivering the intervention and does not include recipient experiences. However, a planned future analysis will provide insights about AYLHIV experiences with the DiSC intervention from a client perspective.

## Conclusion

Given recent policy changes expanding eligibility of DSD to ALHIV, there is limited description of factors impacting implementation of differentiated services for AYLHIV in real world settings. Identifying key facilitators and barriers to implementation of new interventions that incorporate elements of DSD for AYLHIV can inform strategies about how to support risk assessment and service frequency for ALHIV at HIV care facilities across Kenya. Consideration of resource continuity challenges, leadership engagement at multiple levels, integration into clinic workflows, and alignment with changing national guidelines will be important for sustained use and scale-up of the DiSC intervention.

## Supplementary Information

Supplementary Material 1.

## Data Availability

Data cannot be shared publicly because of the sensitive nature of qualitative data. The data are available from the University of Washington for researchers who meet criteria for access to qualitative data.

## Abbreviations

AYLHIV: Adolescents and young adults living with HIV
ART: Antiretroviral Therapy
CFIR: Consolidated Framework for Implementation Research
CQI: Continuous Quality Improvement
DiSC: Data-informed Stepped Care to Improve Adolescent Outcomes Study
DSD: Differentiated Service Delivery
EMR: Electronic Medical Record
FGD: Focus Group Discussion
LMIC: Low and Middle-Income Countries
PHQ-2: Patient Health Questionnaire-2
PHQ-9: Patient Health Questionnaire-9
TCAs: To Come Again (Clinic appointments)
WHO: World Health Organization
