# Supplementary material for: Determinants of implementation of a stepped care intervention for adolescents and youth living with HIV in Kenya: a qualitative evaluation
Source: BMC Health Serv Res. 2025 May 15;25:702. doi: 10.1186/s12913-025-12875-7 (PMC12079965; doi:10.1186/s12913-025-12875-7)
Supplement: Supplementary file 1 — Supplementary Material 1. [file 12913_2025_12875_MOESM1_ESM.docx]

| **DiSC – Data-informed Stepped Care** |
| --- |
| **Healthcare Worker**  **Focus Group Discussion Guide** |

**Semi-Structured Focus Group Discussion Questions**

1. First, can you tell me a little about your clinic/facility, including how things work now, and what the overall climate [climate = energy, feel, environment] is like?

**[Note: This question asks about the clinic generally, and the overall environment and structure at the clinic. It is not specifically asking anything about the Stepped Care intervention at this point]** (CFIR domains: Inner setting and Individuals involved in the implementation)

- - ***Possible probes:*** relationships with supervisors and colleagues, communication structures and processes in the clinic, relationships with others in the clinic, relationships between this clinic and other clinics/facility, perceptions/beliefs on the ability to try new services/processes in the clinic (both individually and collectively as a team), willingness to try new things or change clinic processes (both individually and collectively as a team)

1. Overall, what did you think of the idea of providing Stepped Care to adolescents and young adults at the beginning of the study?

**[Note: This question asks about initial/early implementation of Stepped Care, including training during the first month or two after being trained]**

- - ***Possible probes:*** training delivery and design, initial beliefs about the intervention, overall processes of delivering new intervention services, comparison to previously offered services and processes for offering those services in the facility, initial thoughts on challenges or benefits of providing stepped care, whether they are acceptable (you like and welcome stepped care) and feasible (seems possible or doable to use in your clinic)

1. What is it like now to use the DiSC tool and to provide Stepped Care services during the study?

**[Note: This question asks about implementation of Stepped Care at 6 months post roll-out]**

(CFIR domain: Characteristics of the Intervention)

- - ***Possible probes:*** fidelity (fidelity = implementation as trained) to the study design/planned implementation, beliefs about the intervention, comparison to previously offered services, challenging and positive experiences with providing stepped care, suggestions for improving fidelity (use of DiSC tool for correct assignment to steps, AYA receiving the correct interventions), acceptability (making it something you like/welcome), and feasibility (use of DiSC tool and providing Stepped Care seems possible to do)

1. [*I’d like to learn more about other factors, such as stakeholders and external factors such as policies that may impact implementation of Stepped Care]*

Who supported implementing Stepped Care during the study?

**[Note: This question asks about WHO has had a key role in helping with implementation of Stepped Care in the clinics; some providers may be less aware of buy in from community and country/county leaders]** (CFIR domain: Outer Setting)

- - ***Possible probes:*** Role of the study team, overall distribution of responsibilities within the clinic/facility, leaders within the clinic or facility, influence or buy in from community, county leaders, country leaders

1. What should we be thinking about if we wanted to implement Stepped Care services and integrate it into routine care in other facilities/clinics in Kenya?

**[Note: This question asks about the future of scaling up Stepped Care to other health facilities in Kenya]** (CFIR domain: Process of Implementation)

- - ***Possible probes:*** Beliefs about intervention effectiveness and impact (eg: are healthcare providers able to use the tool to correctly assign AYA to the correct steps, are more AYA retained in care, are AYA receiving the correct interventions to support them in care retention at their facility), beliefs about what would need to be adapted for sustainability (continued use of Stepped Care), beliefs about what would need to happen for scalability (expansion of Stepped Care to other clinics and settings), suggestions for who would need to be involved in scaling to other settings (MOH, clinic leadership, etc)

As we finish talking today, are there any other questions we should be asking people like you to help us understand whether and how the Stepped Care intervention can be implemented at your facility/clinic?

Is there anything about the Stepped Care intervention or the DiSC tool that you think is important to tell me, but I didn’t ask about?

That is all of the questions I have for you today. Thank you for your time. If you do not have any further questions or comments, I will now turn off the recorder*.*
